# P02.188. Integrative health care services as a function of body mass index

**DOI:** 10.1186/1472-6882-12-S1-P244

**Published:** 2012-06-12

**Authors:** N Yang, R Wolever, R Roberts, A Perlman, R Dolor, G Ginsburg, L Simmons

**Affiliations:** 1 Duke Integrative Medicine, Durham, USA; 2 Duke Clinical Research Institute, Durham, USA; 3 Duke Institute for Genome Sciences & Policy, Center for Personalized Med, Durham, USA; 4 Duke University Center for Research on Prospective Health Care, Durham, USA

## Purpose

The prevalence of obesity in the U.S. has become a serious health threat in the last two decades. Despite increased use of integrative medicine (IM) for chronic disease, research on the utilization and effectiveness of IM for management of obesity is limited. The purpose of this study was to: (1) examine how patient reasons for seeking IM care, patient treatment goals, and services provided differs based on body mass index (BMI); and (2) characterize psychosocial functioning, health behaviors, and current medical conditions across BMI classifications in patients seeking IM.

## Methods

We analyzed data from eligible participants in the BraveNet practice-based research network who saw a physician at one of eight IM clinics (N=2,036). Participants self-reported demographics, health behaviors, psychosocial data, their reasons for seeking IM care, and treatment goals. Providers reported medical condition(s) treated and services provided.

## Results

Comparing across BMI categories, more overweight and obese subjects reported decreasing pain as a primary goal of their IM care, while more underweight and normal weight subjects reported seeking improvements in mood. Further, more overweight participants reported seeking IM care because someone they trust recommended the center, while more obese participants reported wanting greater input into their health care decisions. The services most frequently provided to all patients included acupuncture, IM consultation, nutrition services, and preventive care, with significantly greater frequency of preventive care provided to underweight participants. Differences in psychosocial and behavioral factors included: decreased frequency of aerobic exercise and muscular strengthening and increased depressive symptoms, fatigue, and pain with increasing BMI. There were no significant differences in the prevalence of chronic diseases across BMI categories.

## Conclusion

This is the first study to characterize IM patients based upon BMI. Results may lay the groundwork for future studies aimed at tailoring IM strategies for the prevention and management of obesity and comorbidities.

